# Anabolic Androgenic Steroids: Searching New Molecular Biomarkers

**DOI:** 10.3389/fphar.2018.01321

**Published:** 2018-11-20

**Authors:** Francesco Sessa, Monica Salerno, Giulio Di Mizio, Giuseppe Bertozzi, Giovanni Messina, Benedetta Tomaiuolo, Daniela Pisanelli, Francesca Maglietta, Pietrantonio Ricci, Cristoforo Pomara

**Affiliations:** ^1^Department of Clinical and Experimental Medicine, University of Foggia, Foggia, Italy; ^2^Department of Legal, Historical, Economic and Social Sciences, University of Catanzaro, Catanzaro, Italy; ^3^Medical and Surgical Sciences, University of Catanzaro, Catanzaro, Italy; ^4^Department of Medical, Surgical and Advanced Technologies “G.F. Ingrassia”, University of Catania, Catania, Italy

**Keywords:** anabolic androgenic steroids (AASs), adverse effects, miRNAs, doping, new molecular biomarkers

## Abstract

Even if anabolic androgenic steroid (AAS) abuse is clearly associated with a wide spectrum of collateral effects, adolescents and athletes frequently use a large group of synthetic derivatives of testosterone, both for aesthetic uses and for improving performance. Over the last few years, the development of MicroRNA (miRNA) technologies has become an essential part of research projects and their role as potential molecular biomarkers is being investigated by the scientific community. The circulating miRNAs detection as a diagnostic or prognostic tool for the diagnosis and treatment of several diseases is very useful, because with a minimal quantity of sample (peripheral blood), miRNAs are very sensitive. Even more, miRNAs remain stable both at room temperature and during freeze-thaw cycles. These characteristics highlight the important role of miRNAs in the near future as new tools for anti-doping. The article provides a systematic review and meta-analysis on the role of miRNAs as new potential molecular biomarkers of AAS use/abuse. Particularly, this paper analyzed the “miRNA signature” use as biomarkers for health disorders, focusing on the organ damages which are related to ASS use/abuse. Moreover, this review aims to provide a future prospect for less invasive or non-invasive procedures for the detection of circulating miRNA biomarkers as doping assumption signaling.

## Introduction

Even if anabolic androgenic steroid (AAS) abuse is clearly associated with a wide number of collateral effects, adolescents and athletes frequently use a large group of synthetic derivatives of testosterone, both for aesthetic uses and for improving performance (Smurawa and Congeni, 2007; Bailey et al., 2013; Dickinson et al., 2014).

The market for performance enhancing drugs is now huge and constantly increasing. In fact, the phenomenon of doping no longer affects only professional athletes, but also subjects practicing sports activities at the amateur level (Reardon and Creado, 2014).

The adverse events for long-term usage among adolescents are very similar to those of adults, even if doses and duration are significantly less. On the other hand acceleration of pubertal development and early epiphyseal closure, resulting in reduced adult height, are typical adolescent adverse effects; while masculinization of females and acne appears to be more severe in adolescent (Pärssinen et al., 2000; Zitzmann and Nieschlag, 2003).

The most frequently abused androgens are nandrolone, testosterone, stanozolol, methandienone, and methenolone (Pope and Katz, 1994; Evans, 1997a,b). In particular, athletes seem to prefer intramuscular injections over oral formulations (Evans, 1997a; Hoffman et al., 2009). The use of more than one androgen is more frequent than single agents (Pope and Katz, 1994; Evans, 1997a; Hoffman et al., 2009). For example, athletes may resort to masking agents in order to avoid androgen detection. Among them, diuretics are the most common used, because of increasing urinary volume, thus lowering the concentration of urinary detectable substances. However, multi-analytic screening analyses are capable of detecting them, instead desmopressin and glycerol, other masking agents, require more sophisticated methods (Cadwallader et al., 2010; World Anti-Doping Agency, 2014).

The current anti-doping methods consisting in a single time point evaluation, so limited to the standardized detection techniques that developed until that moment. Therefore, these methods will never be up to date with the increasingly sophisticated doping regimens and the ongoing development of new substances. With this aim, WADA elaborated the athlete biological passport (ABP) as the evolution of drug testing techniques (Bucknall et al., 2014). The final goal of ABP was to determine a highly specific profile for each athlete, evaluating some conventional hematological parameters that should be stable over time in the absence of pathologies or doping (Lippi and Plebani, 2011). Nevertheless, to date the fight to the doping remains open and for all these reasons, the identification of new molecular biomarkers remains an ambitious target for the scientific community.

Over the last few years, the development of MicroRNA (miRNA) technologies has become an essential part of research projects and their role as potential molecular biomarkers is being investigated by the scientific community. Main clinical applications of miRNA dosage and deregulation are: (i) cancer characterization and prediction of the course of a disease (Yu et al., 2008; Segura et al., 2010; Salerno et al., 2018); (ii) viral infection diagnosis (Lecellier et al., 2005); (iii) implications in nervous system development (Mehler and Mattick, 2007); (iv) cardiovascular disorder diagnosis (Van Rooij et al., 2006; Hébert and De Strooper, 2009; Ai et al., 2010; Wang et al., 2010); (v) identification of specific patterns in primary muscular disorders (Eisenberg et al., 2007); (vi) differences among diagnosed type 1 diabetes and healthy control diabetes (Nielsen et al., 2012). These associations demonstrate that utilizing these abnormally expressed miRNAs as biomarkers for diseases is a valuable diagnostic strategy.

This article provides a systematic review and meta-analysis on the role of miRNAs as potential new molecular biomarker of AAS use/abuse. Particularly, this paper analyzed the “miRNA signature” use as biomarkers for health disorders, focusing on the organ damages which are related to ASS use/abuse. Moreover, this review aims to provide a future prospect for less invasive or non-invasive procedures for the detection of circulating miRNA biomarkers as doping assumption signaling.

## Mechanisms of androgen action

The anabolic androgenic effects are linked to the androgen receptor (AR)-signaling action. Androgen receptors are expressed in myosatellite cells (also named satellite cells); these are the precursors of skeletal muscle cells (Sinha-Hikim et al., 2004).

There are three main action mechanisms: (i) directly on AR; (ii) via dihydrotestosterone (DHT) produced by the action of 5-a-reductase, and (iii) via estrogen receptors by means of estradiol produced by CYP19 aromatase. In particular, free testosterone is transported into target tissue cell cytoplasm; binding to the AR takes place either directly or after conversion to 5α-dihydrotestosterone (DHT) by the cytoplasmic enzyme 5-alpha reductase. Into the cell nucleus, both free or bound, testosterone acts on specific nucleotide sequences of the chromosomal DNA. The produced mRNA can activate DNA transcription of specific responsive genes (Handelsman et al., 2015).

AAS mimics the testosterone physiological effects, and primarily act via the androgen receptor. However, even if the anabolic action of androgens on the skeletal muscle has been extensively investigated, it is not completely known. Evidence supports the theory that androgens influence differentiation of mesenchymal, multipotent stem cells, promoting myogenic lineage going to the detriment of the adipogenic one (Singh et al., 2003, 2006, 2009).

## Adverse effects of androgen abuse

To date, the AAS use/abuse is frequently linked to widespread serious health damages: indeed, even the cases of a one-time cycle (use over a specific time-period) at very low doses can cause irreversible harmful effects after the cycle is completed. Moreover, recreational users and/or athletes utilize AASs in association with other drugs, such as stimulants and/or depressants. In this way, the correct attribution of adverse effects to AAS use/abuse becomes very difficult. Furthermore, when a side effect occurs in athletes or bodybuilders, it is very difficult to elucidate if the adverse effects were linked to androgen use or to “athlete” status (Pope et al., 2014).

**Figure 1:**
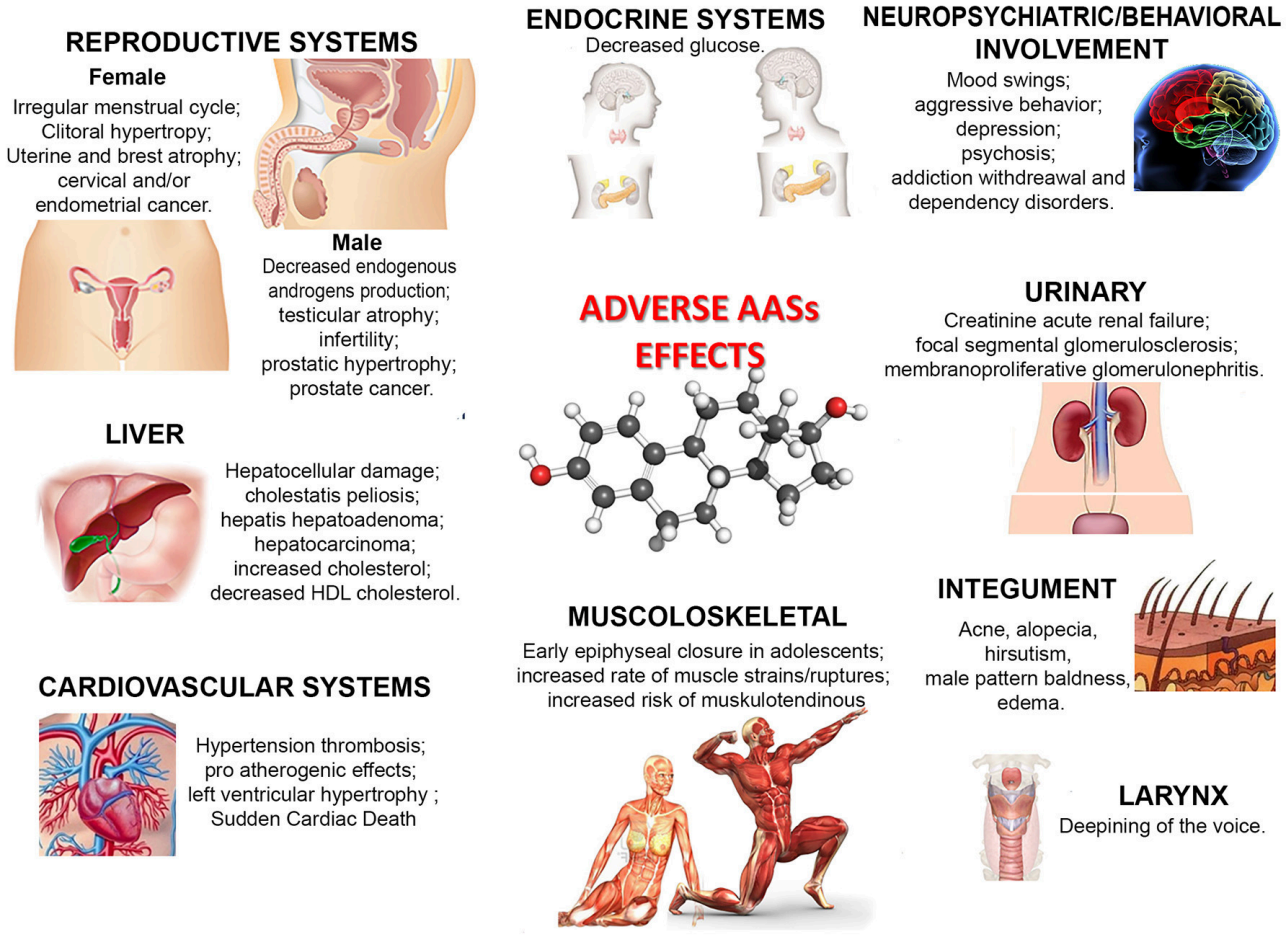
Principal adverse effects of AAS use/abuse.

Nonetheless, as illustrated in Figure 1, a large number of side effects related to AAS use/abuse has been described. The illicit use of AASs provokes or favors the development of serious health pathological conditions, such as hypertension, atherosclerosis, hepatic damages and tumors, tendon ruptures, reduced libido, and psychiatric/behavioral disorders such as aggressiveness and irritability (Stannard and Bucknell, 1993; Yesalis and Bahrke, 1995; Mewis et al., 1996; Bertozzi et al., 2017; White and Noeun, 2017; Junior et al., 2018). The effects on mood and behavior are also well established: depressive, hypomanic or manic episodes, sometimes associated with psychotic symptoms, increased risk of suicidal or homicidal death have been observed in AAS users (Pope and Katz, 1994; Kanayama et al., 2008; Piacentino et al., 2015). Occasional studies reported adverse renal, immunologic and musculoskeletal effects (Kanayama et al., 2010; Pope et al., 2014).

## Circulating MicroRNAs: new molecular biomarkers

**Figure 2:**
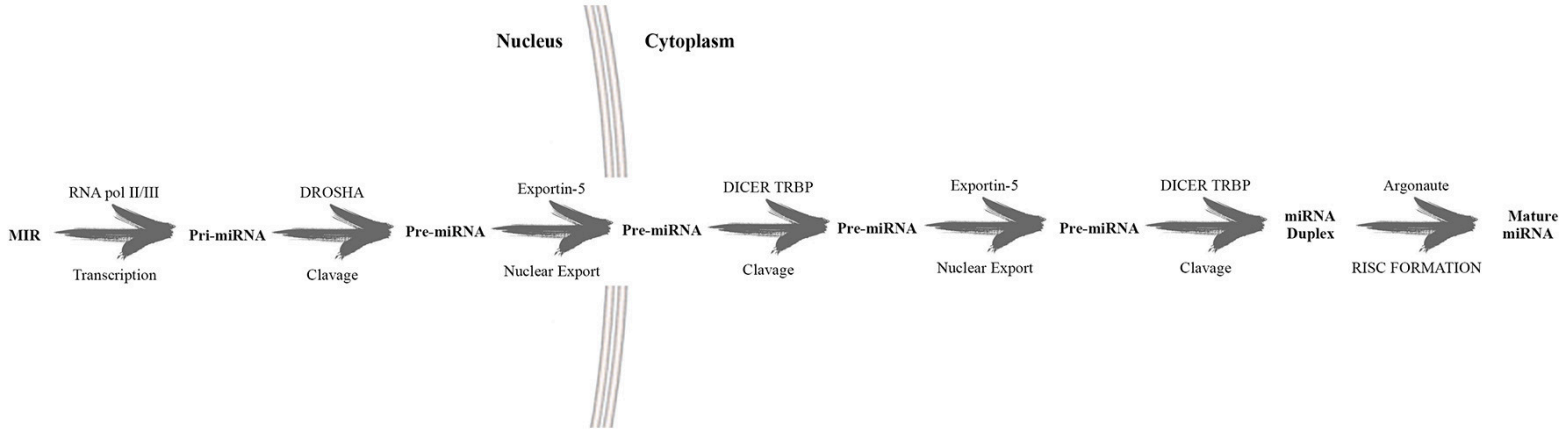
Schematic description of miRNA biogenesis. In the nucleus RNA polymerase II transcribed the long primary miRNA which is later converted by the endonuclease Drosha intopre-miRNA. This pre-miRNA is exported into the cytoplasm and further cleaved by Dicer, developing mature miRNAs.This latter recognizes 3′ untranslated regions, guided by RNA, inducing silencing complexes (RISC). The result is the silencing of target expression.

MicroRNAs (miRNAs) are 20–22 nucleotide non-coding RNA molecules, which regulate gene expression at the post-transcriptional level, located in intergenic or intronic regions as individual or clustered genes (Bartel, 2004). Several steps must occur before miRNAs can act, including the action of many enzymes, such as RNA polymerase II, Drosha, Exportin 5, Dicer and Argonaute (Ago). Figure 2 shows a schematic description of miRNA biogenesis (MacFarlane and Murphy, 2010; Slezak-Prochazka et al., 2010).

miRNA nomenclature was based simply on the sequence of discovery, with few notable exceptions (such as let-7 and lin-4). Subsequently, the identifiers indicating the species (hsa, human; mmu, murine; etc.) were added. Moreover, other symbols (such as “^*^”) or numbers (-5p or 3p) could be added, identifying better the miRNAs (for example indicating the homology, the guide miRNA strand, etc…) (Desvignes et al., 2015).

These small non-coding RNAs regulate gene expression by RNA-RNA interactions, but this is not the only mechanism to control protein production; other mechanisms are: ribosomal RNA modifications, repression of mRNA expression by RNA interference, alternative splicing (Catalanotto et al., 2016).

Emerging evidence demonstrate that serum miRNAs remain stable at different temperature conditions if compared to other source of miRNA. This characteristic is very important because miRNAs could be used to detect illicit substance consumption in the same way that they are used as biomarkers of diseases (Chana et al., 2013).

To date, several papers have described the potential use of circulating miRNAs as specific biomarkers in the anti-doping field. For example, the use of continuous erythropoietin receptor activator induces higher plasma levels of miR-144: the plasma dosage of this miRNA could be used for the detection of erythropoiesis-stimulating agents (Zhou et al., 2015). Another study described a relationship between the expression of 4 miRNAs and the use of recombinant human GH (Keane et al., 2015). Moreover, the study of Leuenberger et al. highlighted the importance of circulating miRNAs as biomarkers for autologous blood transfusions (Leuenberger et al., 2013b). The use of circulating miRNAs to detect performance-enhancing agents could be incorporated into the adaptive model of the ABP, considering their high stability in blood and unmodified characteristics when exposed to the environmental factors (Ponzetto et al., 2016). Furthermore, the possibility to detect miRNAs not only in serum and plasma but also in urine, saliva and other body fluids (Gilad et al., 2008), makes these new molecular biomarkers the new frontier of the fight against doping.

## Circulating MicroRNAs and AAS adverse effects

In the next sections, all side effects related to AAS abuse are analyzed, reporting the candidate miRNAs investigated in previous studies for their possible use as molecular biomarkers (Table 1).

**Table 1:** Organ damage and miRNA expression profiles investigated in the literature.

	**Differentially expressed miRNAs**		**References**
	**Upregulated**	**Downregulated**
**CARDIOVASCULAR SYSTEM AND HEART**			
Ischemia		miR-1 miR-133	Song et al., 2015
Hypertrophy	miR-208a; miR-150; miR-23a; miR-24; miR-21; miR-195; miR-199	miR-1; miR-26b; miR-27a; miR-143; miR-29; miR-133	Hata, 2013; Joladarashi et al., 2014; Wong et al., 2016
Cardiac fibrosis	miR-21; miR-133	miR-29	Joladarashi et al., 2014
Arrhythmia	miR-1; miR-133; miR-133a; miR-212;miR-17- miR-92;miR-106bb-; miR-25	miR-150	Joladarashi et al., 2014
**MUSCULOSKELETAN**			
After 7 days of mechanical overload		miR-1; miR-133	Kirby et al., 2015
Slow twich fibers	miR-208b; miR-499		Kirby et al., 2015
**REPRODUCTIVE SYSTEM DISEASE (MALE)**			
Prostate cancer	miR-200c, miR-20a, miR-20b, miR-182	miR-222, miR-221, miR-145, miR-214, miR-125b, miR-143, miR-29a, miR-24, miR-199a	Afshar et al., 2014
	miR-375; miR-17; miR-93; miR-106a; miR-141; miR-720; miR-7a; miR-200b; miR-21; miR-106b; miR-375; miR-663b; miR-615-3p; miR-425-5p; miR-663a; miR-182-5p; miR-183-5p	miR-205-5p; miR-221-3p; miR-222-3p; miR-376c-3p; miR-136-5p; miR-455-3p; miR-455-5p; miR-154-5p	Kristensen et al., 2016
	miR-let-7a-2, miR-let-7i, miR-16-1, miR-17-5p, miR-20a, 21, miR-24-1, miR-25, miR-27a, miR-29a, miR-29b-2, miR-30c, miR-32, miR-34a, miR-92-2, miR-93-1, miR-95, miR-101-1, miR-106a, miR-124a-1, miR-126a-1, miR-135-2, miR-146, miR-149, miR-181b-1, miR-184, miR-187, miR-191, miR-196-1, miR-197, miR-199a-1, miR-214, miR-128a, miR-195, miR-198, miR-199a-1, miR-199a-2, miR-203, miR-206, miR-2014, miR-2018-2, miR-223, miR-202, miR-210, miR-296, miR-320, miR-370, miR-373, miR- 498, miR-503	Let 7a, let- 7b, let-7c, let-7d, let-7g, 16, 23a, 23b, 26a, 92, 99a, 103, 125a, 125b, 143, 145, 195, 199a, 221, 222, 497	Volinia et al., 2006; Porkka et al., 2007
	miR-Let-7a-5p, miR-let-7d-3p, miR- let-7d-5p, miR- 7b-5p, miR-20a- 5p, miR-21-3p, miR-25-3p, miR-29b-2-5p, miR-30d-3p, miR-92a-3p, miR-92b-3p, miR-93-3p, miR-96-5p, miR-103b-3p, miR-182-5p, miR-183-5p, miR-375, miR-421, miR-423-3p, miR-423-5p, miR- 425-5p, miR-484, miR-615-3p, miR-663a, miR-663b, miR-664a-3p, miR-1248, miR-1260a		Kristensen et al., 2016
**REPRODUCTIVE SYSTEM DISEASES (FEMALE)**			
POF (Blood)	miR-202; miR-146a; miR-125b-2; miR-139-3p; miR-654-5p; miR-27a; miR-765; miR-23a; miR-342-3p; miR-126	miR-Let-7c; miR-144	McGinnis et al., 2015
Follicle atresia	miR-936; miR-26b; miR-149; miR-10b; miR-574-5p; miR-149; miR-1275; miR-99a	miR-Let-7i; miR-92b; miR-92a; miR-1979; miR-1308; miR-1826	Li M. et al., 2015
Ovarian cancer	miR-21; miR-203; miR-205	miR-200 (ovarian cancer cell migration)	Donadeu et al., 2017
**CENTRAL NERVOUS SYSTEM**			
Depression (LH)	miR-96, miR-141, miR182, miR-183, miR-183*, miR-198, miR-200a, miR-200a*, miR-200b, miR-200b*, miR-200c, and miR-429.		Dwivedi, 2014
Non-depression (NLH)		miR-96, miR-141, miR182, miR-183, miR-183*, miR-198, miR-200a, miR-200a*, miR-200b, miR-200b*, miR-200c, and miR-429.	Dwivedi, 2014
**LIVER**			
Hepatic hypercholesterol and hyperlipid metabolism	miR-122; miR-21; miR-23		Szabo and Bala, 2013
Inflammatory response Hyperinflammation	miR-155, miR-132, miR-125b, miR-146a, miR-150, miR-181, let-7 and miR-21.20,21		Szabo and Bala, 2013
drug-induced liver injury (DILI)	miR-710 and miR-711 miR-16a, miR-328 and miR-299-5p	miR-122 and miR-192 miR-122a	Szabo and Bala, 2013
**KIDNEY**			
kidney fibrosis	miR-21		Badal and Danesh, 2015
renal fibrosis, tubular hypertrophy, glomerular alterations	miR-200a, miR-200b, miR-141, miR-429, miR-205, and miR-192.		Wei et al., 2013
Acute kidney injury (AKI)	mir-21, mir-205, mir-127 and mir-494		Wei et al., 2013
**SKIN**			
Dermatomyositis	miR-146b and miR-155	miR-1, miR-133, miR-206, miR-11040; miR-30a-3p	Luo and Mastaglia, 2015

### Cardiovascular system and heart

The adverse effects on the heart and cardiovascular system caused by AAS abuse have been frequently investigated: harmful changes in the risk factors for cardiovascular pathology are frequently described, such as the decrease in plasma HDL cholesterol levels (Payne et al., 1985; Glazer, 1991; Daly et al., 2003; Frati et al., 2015a) and changes in clotting factors (Ansell et al., 1993). Moreover, perhaps through a direct cardiotoxic action, which results in cardiomegaly and cardiovascular failure, AASs are often involved in the sudden cardiac death of young consumers (Frati et al., 2015b; Albano et al., 2017; Sessa et al., 2018). Nevertheless, the relation between AAS use/abuse and cardiovascular adverse effects should be clarified (Corona et al., 2014; Morgentaler et al., 2015).

MiRNAs exert their action though signaling and transcriptional pathways affecting cardiac development, function, and disease. With the aim of identifying miRNAs related to dysfunctions, several heart diseases (Ischemia, Hypertrophy, cardiac fibrosis, and Arrhythmia) have been investigated.

MyomiRs (miR-208a, miR-208b and miR-499) play a pivot role in the control of myosin heavy chain isoform expression (Van Rooij et al., 2009), while miR-1 and miR-133a carry out important functions in cardiac differentiation and development (Liu and Olson, 2010; Humphreys et al., 2012). miR-133a is associated with a large number of cardiac pathologies; miR-1 expression in the ischemic zone was found to be significantly increased (Yang et al., 2007). Moreover, miR-1 and miR-133 increased the risk of arrhythmia in the ischemic heart (Huang et al., 2011; Song et al., 2015). Several studies reported elevated plasma levels of miR-1, miR-133, miR-208a, and miR-499, after acute myocardial infarction, suggesting a role as valuable prognostic biomarkers (Hata, 2013; Joladarashi et al., 2014; Wong et al., 2016).

### Musculoskeletal apparatus

Skeletal muscle represents the example of the tissue subjected to environmental impulses (nutrients and exercise) and, about that, AAS use is frequently combined with resistance training. This practice is linked to a higher risk of tendon injury (Seynnes et al., 2013). Moreover, when the use/abuse occurs in adolescents, a premature closure of the epiphyses could take place, reducing the influencing the height (Schultzel et al., 2014).

Several studies using northern blot analyses reported that the myomiR family (miR-1, miR-133a, miR-133b, miR-206, miR-208a, miR-208b, and miR-499) is strictly striated-muscle specific (Sempere et al., 2004; McCarthy, 2008; Van Rooij et al., 2009; Zhang et al., 2016).

Muscle fibers are typically distinguished in type I (slow) and type II (fast) fibers. miR-208b and miR-499 could play an important role in inhibition of fast myofiber genes and promotion of slow myofiber genes (Nie et al., 2015). Considering that AASs are strictly related to muscular hypertrophy, a direct relationship to these miRNA levels expressed in plasma and drug abuse could be hypothesized.

It is interesting that after training the mature levels of miR-1, miR-206 and miR-133 decrease both at the tissue level and the plasma level (Kirby et al., 2015). Furthermore, miR-206 plasma levels were reported to be up-regulated in aged human skeletal muscle (Kim et al., 2014).

At the light of these findings, a direct relationship between miRNA plasma levels and type of sport practiced could be considered, given that frequently AAS use is associated with these activities.

### Reproductive systems

AASs are related to various side effects in males (hypofertility and gynecomastia) and in females (virilization and hirsutism, acne, irregular menses, lower-pitched voice, and male-pattern baldness, increased body hair and sex drive) (Evans, 1997a; Parkinson and Evans, 2006; Zahnow et al., 2017).

In males, moreover, there are multiple effects on the neuroendocrine system such as hypogonadism (especially following abrupt discontinuation of the drug), impotence, suppression of spermatogenesis and inhibition of the hypothalamic–pituitary– testicular axis (Dickerman et al., 1999; Pertusi et al., 2001; Campion et al., 2012; Rahnema et al., 2014; Pomara et al., 2016).

Infact, androgens play a pivotal role in development and maintenance of the male reproductive system; the exogenous administration of androgens leads to a reduction in endogenous production, causing testicular atrophy, androgen deficiency, and infertility (Fronczak et al., 2012; Pomara et al., 2016). Moreover, AAS abuse promotes prostatic hypertrophy and increases the risk of prostate cancer (El Osta et al., 2016; Albano et al., 2017).

Aberrant expression of numerous miRNAs was reported related to different reproductive system diseases. For example, the expression profile of miR-203 was found to be altered in prostate imbalance (Bucay et al., 2015). Another study described a pivotal role for miR-93 and miR-648 in prostate cancer regulation (Zhang et al., 2014). Afshar et al. (2014) reported a set of 23 miRNAs with expression changes (17 overexpressed and 6 underexpressed). Notwithstanding the numerous studies, to date it is very difficult to establish which are the miRNAs that could be considered as important molecular biomarkers for these diseases (Bertoli et al., 2016; Luu et al., 2017).

The principal adverse effects derived from AAS use on the female reproductive system are related to the subtle equilibrium of the female hypothalamus-pituitary-gonadal axis, which becomes twisted consequently to increases in concentration of circulating testosterone and reduction in estrogen activity. In particular, the diminishing in estrogen and progesterone levels results in: (i) inhibition of follicle formation; (ii) ovulation, (iii) irregular menstrual cycle, (iv) amenorrhea (absence of the menstrual cycle). Some authors, in addition, reported a very high risk of development of cervical and/or endometrial cancer, uterine atrophy, and, then, infertility (Sarojini et al., 2012).

As stated before, circulating miRNAs are considered as possible biomarkers for the identification of different pathological conditions. miRNA-9, miRNA-18b, miRNA-32, miRNA-34c, and miRNA-135a were reported to be significantly increased in polycystic ovary syndrome (McGinnis et al., 2015). Other studies reported that several miRNAs are upregulated (hsa-miR-10b, hsa-miR-26b, hsa-miR-99a, hsa-miR-149^*^, hsa-miR-574-5p, Hsa-miR-936, hsa-miR-1275, mmu-miR-1224, P-miR-466 g-b, P-miR-1275, P-miR-1281, R-miR-26b) in cases of follicle atresia and others downregulated (hsa-let-7i, hsa-miR-92a, hsa-miR-92b, hsa-miR-1308, hsa-miR-1826, hsa-miR-1979, P-miR-923, P-miR-1826, R-let-7a, R-miR-739, ssc-miR-184) (Li Y. et al., 2015).

It is very interesting that miR-21, miR-203, and miR-205 showed higher levels in ovarian cancer compared to normal ovary state, suggesting an important role as molecular biomarkers (Donadeu et al., 2017).

Moreover, in endometriosis a dysregulation of several blood miRNASs was reported: upregulation of miR-122 and miR-199a levels while downregulation of miR-9 ^*^, miR-141 ^*^, miR-145 ^*^, miR-542-3p (Marí-Alexandre et al., 2016).

### Central nervous system

The side effects linked to AAS use on the central nervous system (CNS) are prevalently linked with the behavioral sphere such as impulsive behavior, aggression, anxiety, hypomania, and occasionally, depressive disorders (Henderson et al., 2006; Hildebrandt et al., 2014). Moreover, miRNAs are expressed highly in neurons, and neuronal miRNA pathways can create an extremely powerful mechanism to dynamically adjust the protein content of neuronal compartments.

However, miRNAs are hypothesized to play a specialized role in cellular responses to stress (Hunsberger et al., 2009), indeed acute stress induces in specific encephalic areas the transient increase in the expression of several miRNAs (Let-7 a-e, miR-9, miR-9^*^, miR-26b, miR-29b, miR-30b, miR-30c, miR-30e, miR-125a, miR-126-3p, miR-129-3p, miR-207, miR-212, miR-351, miR-423, miR-487b, miR-494, miR-690, miR691, miR-709, miR-711).

As a peripheral biomarker in major depressive disorders, miRNAs that have been described as dysregulated are miR-107, miR-133a, miR-148a, miR-200c, miR-381, miR-425-3p, miR-494, miR-517b, miR-579, miR-589, miR-636, miR-652, miR-941, and miR-1243. Among them, only two miRNAs, miR-589 and miR-941, showed stable overexpression in depressive disorders (Dwivedi, 2014). Moreover, two of the best-studied miRNAs related to cognitive impairment roles are miR-124 and miR-137. An up-regulation of miR-124 was associated with an improvement in learning and memory, while miR-132 plays an important role in neurogenesis: these two miRNAs could be used as molecular biomarkers for brain functionally and pharmacological therapies (Shi, 2014; Nadim et al., 2017; Simion et al., 2017).

A controversial role is played by miR-128: several reports in the literature describe a different behavior according to different biological conditions, resulting in some instances of up-regulation whereas, in some, it is down-regulated (Adlakha and Saini, 2014).

### Liver

Several adverse effects on the liver are related to AAS use/abuse, such as hepatic peliosis, cholestatic jaundice and hepatic neoplasms. This latter pathology was not clinical relevant during subject lifetime, but it was diagnosed only during autopsies (Søe et al., 1994; Neri et al., 2011; Turillazzi et al., 2011).

After liver injury, studies have documented the presence of miRNAs in the circulation. Liver damage caused by chemical toxins and diet can produce a release of various miRNAs inside exosomes, microvescicles, HDL, apoptotic bodies and proteins, in the same way as other disturbances (such as alcohol, acetaminophen, viral, or bacterial infection, etc…) (Szabo and Bala, 2013). miR-122 has a central role in the control of lipid metabolism. MiR-16, miR-33, miR-34, miR-103, miR-104, and miR-370, instead, are involved in lipid metabolism too: their serum levels are significantly higher in patients with non-alcoholic fatty liver disease compared to controls (Takahashi et al., 2013; Calvopina et al., 2016; Nelson Hayes and Chayama, 2016).

Moreover, a large number of miRNAs have been related to inflammatory responses (let-7, miR-21.20,21, miR-125b, miR-132, miR-146a, miR-150, miR-155, and miR-181). As previously described, serum levels of miR-122, miR-132, and miR-155, seem to be related to HCV infection, and they were evaluated as markers of inflammation (Szabo and Bala, 2013; Takahashi et al., 2013).

Many studies on miRNAs involved in liver injury caused by drug assumption were evaluated: in serum samples miR-122, miR-125b, miR-146a, miR-155, and miR-192, were up-regulated (Bafunno et al., 2012; Takahashi et al., 2013; Calvopina et al., 2016; Nelson Hayes and Chayama, 2016). Higher level of miR-122 into the bloodstream were documented after hepatocyte death: the plasma levels of this miRNA are upregulated in alcoholic and non-alcholic liver diseases, and virus related consequences (chronic HBV and HCV) (Takahashi et al., 2013; Calvopina et al., 2016).

As previously described, miR-122 is involved in several side effects related to AAS abuse: for this reason, it could be considered as an important molecular biomarker (Zheng et al., 2016).

### Kidney

The mechanisms involved in renal injured after AAS use/abuse can occur in a direct manner, after oral consumption, or an indirect manner, caused by elevated bile salts in plasma (Van Slambrouck et al., 2013).

In some cases, anabolic abuse has been related to the nephrotic syndrome and focal segmental glomerulosclerosis (Herlitz et al., 2010). As previously reported miR-193a (upregulated) and miR-31 (downregulated) are related to focal segmental glomerulosclerosis; moreover, among several miRNAs analyzed in renal fibrosis, miR-21 (upregulated) and miR-22 (downregulated) expression were found altered (Badal and Danesh, 2015).

Other studies described a large number of miRNAs (miR-141, miR-192, miR-200a, miR-200b, miR-205, miR-429) with an increased expression profile in human nephrosclerosis biopsies (Wei et al., 2013).

Several studies described an altered miRNA expression profile in urinary exosomes in diabetic nephropathy patients (miR-130a and miR-145 upregulated, while miR-155 and miR-424 under-regulated) (Li et al., 2014; Li M. et al., 2015).

### Skin

Acne vulgaris and folliculitis are frequently described in AAS users: these symptoms are related to hypertrophy of the sebaceous glands. These side effects usually stop at the end of AAS use. Other side effects are related to the methods of administration: intramuscular injections have been associated with severe infection, such as necrotizing myositis (Hughes and Ahmed, 2011; Zomorodian et al., 2015).

In cases of dermatomyositis, an abnormal expression of miRNAs was detected: with upregulation of miR-146b and miR-155, and downregulation of miR-1, miR-30a-3p, miR-133, miR-206, and miR-11040 (Luo and Mastaglia, 2015).

## Conclusion

The identification of new tools for AAS use/abuse represents an important challenge for the scientific community. In the last few years, several studies have highlighted the role of miRNAs as a highly accurate diagnostic tool. To date, the limits of traditional techniques for diagnosis of numerous diseases (such as cardiac imaging) are costs and being not quantitative. The detection of circulating miRNAs could go beyond these limitations as diagnostic or prognostic tools of several diseases, both because miRNAs are very sensitive, and their detection requires minimal peripheral blood. Several advantages are linked to the use of circulating miRNAs as anti-doping methods: high stability during transport and storage, the long period for detection, not sensitivity to unregulated room-temperature storage, and the stability in plasma subjected to multiple freeze-thaw cycles (Leuenberger et al., 2013a).

**Figure 3:**
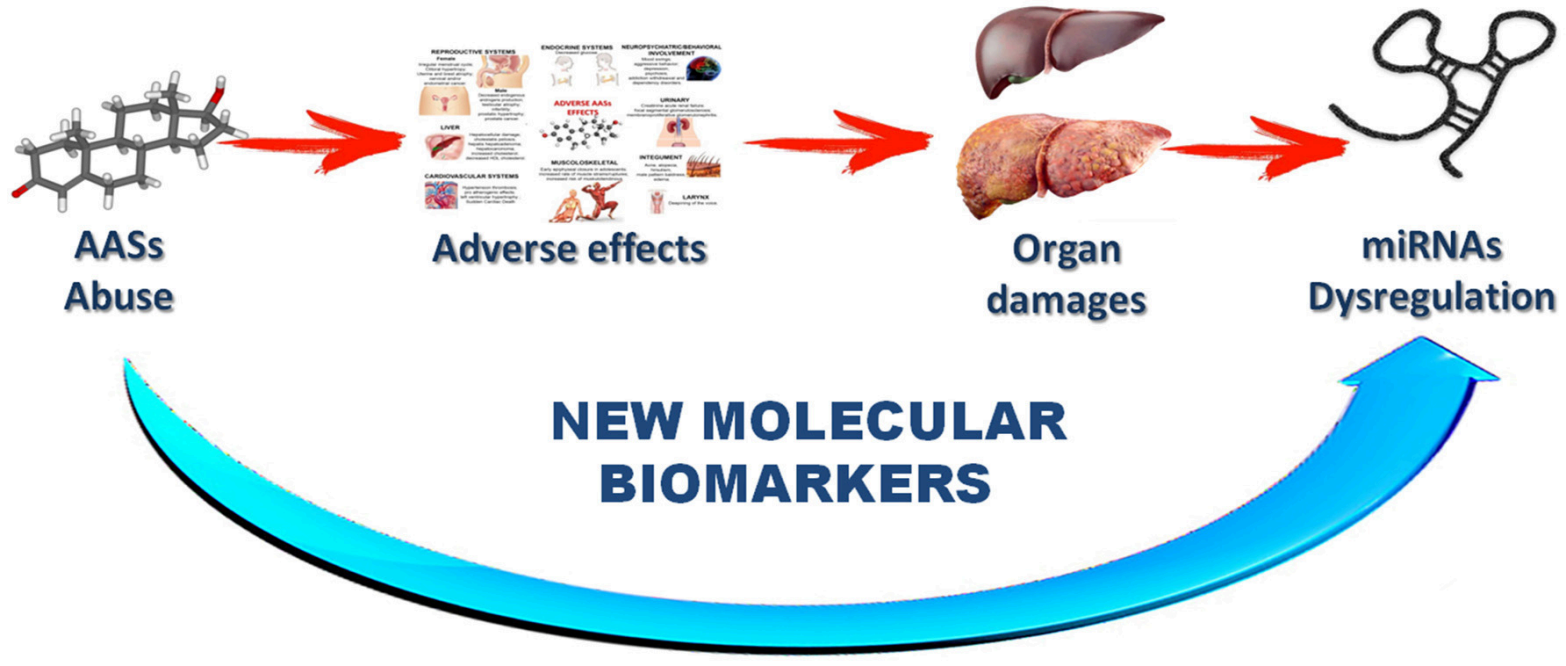
Schematic description of the research hypothesis suggested with this review.

These characteristics highlight the important role of miRNAs in the future as new tools in the anti-doping fight. The research hypothesis of this review is a direct implication among drug assumption, side effects, organ damage and miRNA dysregulation (Figure 3).

At the light of this revision work, a pivot role could be played by miRNAs that seems dysregulated in the cardiovascular or liver diseases, because these organs are interested by adverse effects after AASs use/abuse. For example, it could be very important performed experimental work dosing MyomiRNAs that are a subset of muscle-specific miRNAs. This group comprises a discrete number of molecules (miRNA 1, miRNA 29-b, miRNA 133, miRNA 181-a, miRNA 206, miRNA 208 and miRNA 451). Among these, miRNA 133-a and miRNA 206 were found to be the most promising for understanding the biological response to physical activity and for the potential use for diagnosing muscle injury and in anti-doping testing (Danese et al., 2017). Moreover, performing new experimental studies based on the dosage of miRNAs involved in the control of lipid metabolism (such as MiR-122, MiR-16, miR-33, miR-34, miR-103, miR-104, and miR-370), new tools for a modern anti-doping fight could be obtained.

For example, Salamin et al. (2016) identified three potential candidate miRNAs for testosterone use, even if one of these showed a response related to dose-effect: in fact, levels of miR-122 increased 3.5-fold after 1 day of drug intake. These results suggest that miR-122 could be used as a reliable fingerprint of testosterone misuse. As described in the previous paragraph, this miRNA is strictly related with liver dysfunction: therefore, we could consider a direct involvement as a liver side effect after AAS assumption.

Knowledge regarding miRNAs in human diseases related to AAS use/abuse may eventually lead to identify serum or tissue biomarkers with anti-doping potential. In this regard, the need for careful validation of diagnostic miRNA candidates in well-annotated toxicological studies is mandatory. The rapid progress in anti-doping technologies using miRNA based strategies for the discovery of drug-abuse, such as AAS use/abuse, could optimize new approaches based on existing and emerging knowledge.

## Author contributions

FS and CP contributed to the conception of the study. FS, MS, GD, GB, GM, BT, DP, FM, PR, and CP contributed significantly to literature review and manuscript preparation. FS, MS, GB, and GM wrote the manuscript. FS, GD, GB, FM, and CP helped perform the analysis with constructive discussions. FS, MS, and CP approved the final version.

### Conflict of interest statement

The authors declare that the research was conducted in the absence of any commercial or financial relationships that could be construed as a potential conflict of interest. The handling editor declared a shared affiliation, though no other collaboration, with one of the authors, CP, at time of review.
